# Identification of chilling-responsive microRNAs and their targets in vegetable soybean (*Glycine max* L.)

**DOI:** 10.1038/srep26619

**Published:** 2016-05-24

**Authors:** Shengchun Xu, Na Liu, Weihua Mao, Qizan Hu, Guofu Wang, Yaming Gong

**Affiliations:** 1 Institute of Vegetables, Zhejiang Academy of Agricultural Sciences, Hangzhou, 310021, China; 2 Center of Analysis and Measurement, Zhejiang University, Hangzhou, 310058, China; 3 Department of Life Science, Yuanpei College, Shaoxing University, Shaoxing 312000, China

## Abstract

Chilling stress is a major factor limiting the yield and quality of vegetable soybean (*Glycine max* L.) on a global scale. In the present study, systematic identification and functional analysis of miRNAs under chilling stress were carried out to clarify the molecular mechanism of chilling resistance. Two independent small RNA libraries from leaves of soybean were constructed and sequenced with the high-throughput Illumina Solexa system. A total of 434 known miRNAs and 3 novel miRNAs were identified. Thirty-five miRNAs were verified by qRT-PCR analysis. Furthermore, their gene targets were identified via high-throughput degradome sequencing. A total of 898 transcripts were targeted by 54 miRNA families attributed to five categories. More importantly, we identified 51 miRNAs differentially expressed between chilling stress and control conditions. The targets of these miRNAs were enriched in oxidation-reduction, signal transduction, and metabolic process functional categories. Our qRT-PCR analysis confirmed a negative relationship among the miRNAs and their targets under chilling stress. Our work thus provides comprehensive molecular evidence supporting the involvement of miRNAs in chilling-stress responses in vegetable soybean.

Vegetable soybean (*Glycine max* L.) is a food-type soybean^1^. Largely due to its higher nutritional content and better flavor compared to grain soybean, vegetable soybean has become very popular in China (300,000 ha planted each year), Southeast Asia, and some Western countries^2^,^3^,^4^. However, chilling stress affects vegetable soybean plant growth and results in severe yield reduction^5^. In China, almost 80% of vegetable soybean is planted in spring (from March to April). The seedlings are extremely sensitive to damage from late spring cold temperatures (below to 10 °C), which result in serious reduction of yield, with a decline of more than half in the hardest hit areas. Therefore, understanding resistance mechanisms to chilling stress could promote vegetable soybean yield in agricultural settings. A series of experiments have revealed some physiological processes and molecular mechanisms of soybean under chilling stress, including changes in antioxidant enzyme activity, photosynthesis, and endogenous hormones, as well as DNA and histone modification patterns for resistance to chilling stress^6^,^7^,^8^,^9^,^10^. However, the regulation mechanisms of these processes, especially at the transcriptional and post-translational levels, remain relatively unclear in vegetable soybean.

MicroRNAs (miRNAs), a group of small endogenous noncoding RNAs, play significant roles in post-transcriptional processes by degrading mRNA or inhibiting its translation in plants^11^,^12^. Although most known miRNAs are involved in regulating developmental processes^13^,^14^,^15^, a group of miRNAs have been identified to function under chilling stress in *Arabidopsis thaliana*^16^, *Populus*^17^, and *Prunus persica*^18^. To date, a number of miRNAs have been identified in soybean as responsive to abiotic and biotic stresses, including drought stress, salinity stress, phosphate starvation, and mosaic virus (SMV) and cyst nematode (SCN) infection^19^,^20^,^21^,^22^,^23^. Besides these, several miRNAs from nitrogen-fixing nodules in soybean, e.g. gma-miR166, gma-miR171, and their targets have been identified to have significantly differential expression under chilling stress^24^. However, our knowledge of the roles played by miRNAs under chilling stress conditions is limited, especially for the seedlings of vegetable soybean. A systematic study on chilling-responsive miRNAs is therefore an important step to understanding small RNA-mediated gene regulation in vegetable soybean.

Soybean miRNAs and their targets have been successfully studied by computational and direct cloning approaches^25^, as well as with high throughput sequencing technologies^26^. In the present study, we constructed two independent small RNA libraries, representing chilling stress and control conditions, from vegetable soybean leaves. The high-throughput Illumina Solexa system was employed to identify known and novel miRNAs, as well as their expression patterns. Furthermore, degradome sequencing, which has been successfully confirmed to validate large-scale discovery of miRNA targets^27^,^28^, was used to validate the targets of these detected miRNAs. The aims of this study were 1) to identify conserved and novel miRNAs and their targets in vegetable soybean; 2) to reveal the relationships between miRNAs and their targets under chilling stress. The results provide insights into the regulatory roles of miRNAs under chilling stress in vegetable soybean.

## Results

### Deep sequencing of small RNAs in vegetable soybean

**Figure 1:**
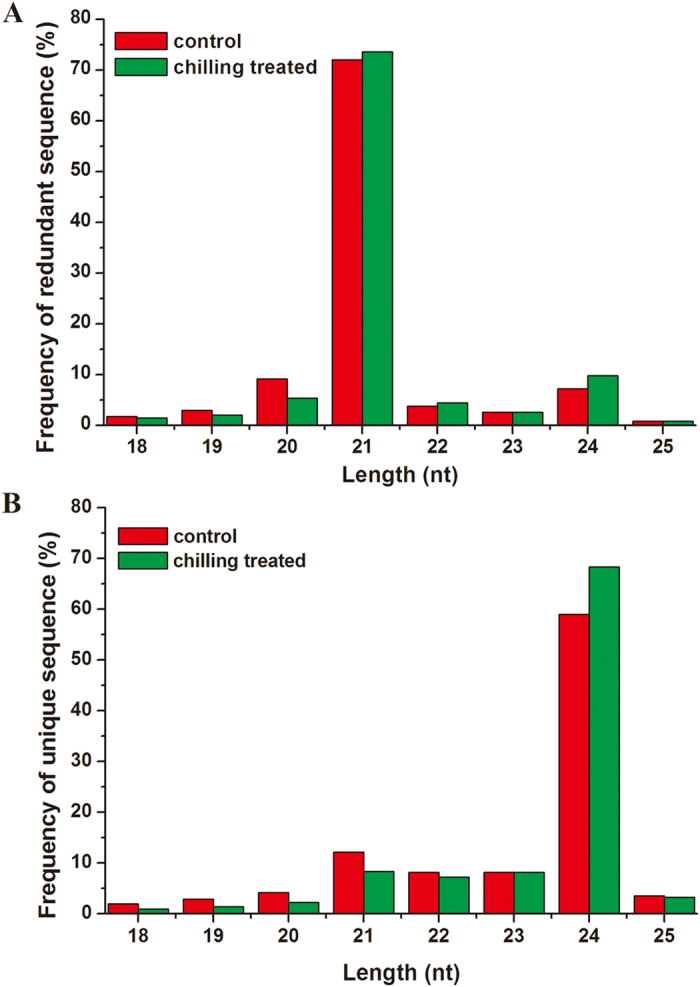
Sequence length distribution of small RNA in chilling and control libraries of vegetable soybeans.

Firstly, the relative growth rate (RGR)^29^ and MDA content^8^ were determined for seedlings kept under control conditions or treated with chilling at 4 °C for 3 h, 6 h, 9 h, 12 h, 24 h and 36 h. At 24 h, there were significant differences in both RGR and MDA content between chilling treatment and the control (Fig. S1). Therefore, leaf samples from the 24-h time point of chilling treatment and the control were used to prepare small RNA libraries. A total of 10,723,343 and 8,944,338 raw sequences were generated from chilling-treated and control libraries, respectively (Table 1). The sequencing data have been deposited into the NCBI/GEO database with accession number GSE76435. After removal of low quality and adapter sequences, there were 8,273,245 and 6,559,098 clean reads from chilling treated and control libraries, respectively. The majority of the redundant reads were 21 nt in length (Fig. 1A), similar to the results of a previous study of *G. max* under drought, salinity and alkalinity stress^30^. The length distribution of the unique sequences showed that the most abundant sequences were 24 nt, accounting for approximately 59.0% and 68.4% of control and chilling-treated libraries, respectively (Fig. 1B).

### Identification of known miRNAs in vegetable soybean

To identify known miRNAs in vegetable soybean, we compared all of the small RNA sequences that we could map with annotated plant miRNAs in miRBase (20.0). Based on sequence similarity, a total of 434 vegetable soybean miRNAs, representing 133 families were identified in the present study (Table S1). Generally, miRNAs consisted of both conserved and species-specific miRNAs. Conserved miRNAs are known to have important functions in plant development and stress response^31^,^32^. We conducted homology searches in a range of plant species to identify miRNA family homologs in vegetable soybean (Table S2). Some identified miRNA families such as miR156, miR160, miR164, miR166, miR396, and miR397 are highly conserved in many plant species, such as *Arabidopsis thaliana*, *Oryza sativa*, and *Zea mays*. In addition, we found several known but non-conserved miRNAs, such as miR3522, indicating that they may have been involved in soybean speciation.

Most of the known miRNAs (63.5%) were 21 nt in length. The miRNAs had a very broad range of expression, ranging from millions of sequence reads to fewer than 10 sequence reads (Table S1). Most of these known miRNAs, such as miR3522, miR1510, miR408, miR1509, miR4996, miR1508, miR1507 and miR167, were relatively highly expressed in both the control and chilling-treated libraries. For example, miR3522 had 3,391,613 and 4,866,673 reads in control and chilling-treated libraries, respectively, which was the most abundant in each of the two libraries. However, some miRNA families had relatively low expression (less than 10), e.g. miR1523, miR1528, miR4366, and miR4389.

### Identification of novel miRNAs in vegetable soybean

**Figure 2:**
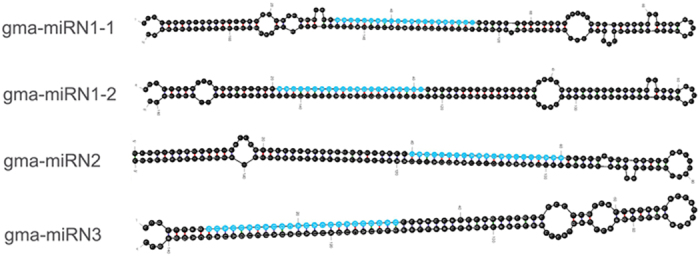
Predicted RNA secondary structure of hairpin-forming precursors of gma-miRN1-1, gma-miRN1-2, gma-miRN2 and gma-miRN3. The putative mature miRNA sequences are shaded in blue. Nucleotide positions are numbered starting from the 5′ end of the precursor sequence.

High-throughput sequencing is often used to detect novel miRNAs in the small RNA transcriptome^33^. For this purpose, we used flanking sequences of candidate miRNAs to predict their hairpin structures based on the soybean genome sequence. Three putative novel miRNA candidates, which could fold into secondary structures, were detected (Table S3, Fig. 2). The negative folding free energies of these pre-miRNA hairpin structures ranged from −49.8 to −80.3 kcal mol^−1^ with an average of about −66.5 kcal mol^−1^, and the minimal folding free energy index (MFEI) ranged from 0.9 to 1.4 with an average of 1.15. However, the expression levels of these novel miRNAs were low, generating fewer than 100 reads, which represented lower expression levels than those of conserved miRNAs.

### Confirmation of predicted miRNAs in vegetable soybean

**Figure 3:**
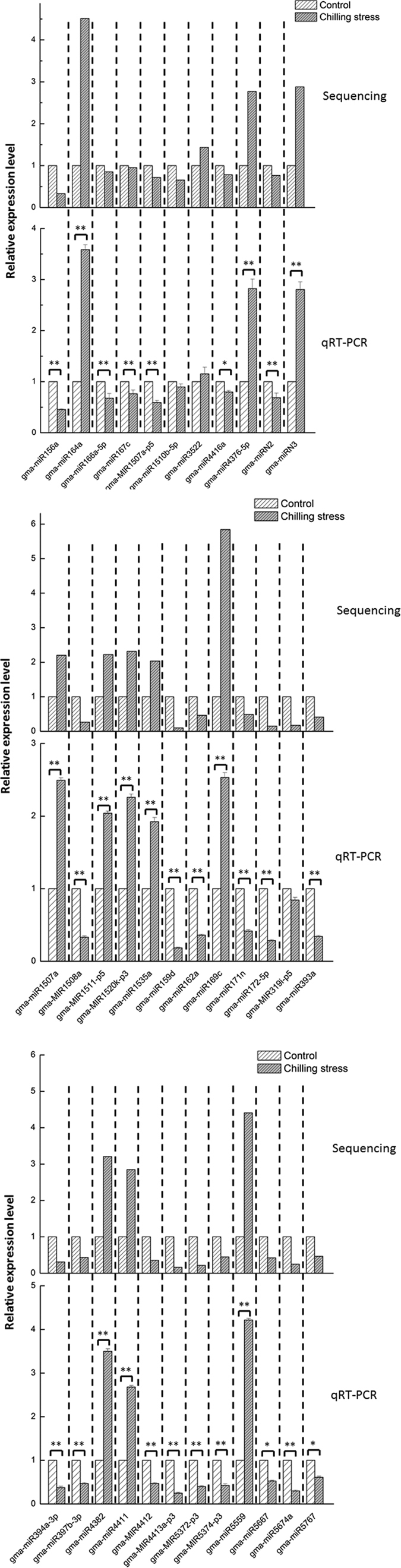
Expression analysis of miRNAs in vegetable soybean by qRT-PCR. Expression was normalized by the level of *U6* in qRT-PCR. All reactions of qRT-PCR were repeated three times for each sample and subjected to an analysis of variance (ANOVA) and the results presented as the mean ± SD. A *P*-value was considered to be statistically significant with (*p < 0.05) or (**p < 0.01).

To validate our sequencing results, we performed quantitative reverse transcription PCR (qRT-PCR) to analyze the expression of the miRNAs. Based on the high-throughput sequencing results, thirty-five miRNAs (including 33 known miRNAs and 2 novel miRNAs), representing different expression levels of sequencing data, were used for qRT-PCR analysis. Overall, expression patterns of these selected miRNAs obtained by qRT-PCR were consistent with the sequencing results (Fig. 3). From linear regression analysis [(miRNA-Sequencing value) = a (RT-PCR value) + b] the correlation coefficient was 0.8048, indicating a positive correlation between RNA-Seq data andqRT-PCRdata (Fig. S2). Although the results showed that the exact fold changes varied between qRT-PCR and miRNA-sequencing data, possibly due to differences in the sensitivity and specificity between the two approaches, the miRNA expression trends are similar. This indicates that the miRNA-sequencing data here are valuable.

### Target validation for vegetable soybean miRNAs

Accurate validation of miRNA targets is important to elucidate potential biological functions of miRNAs^34^. In the present study, we applied high-throughput degradome sequencing technology to identify the targets cleaved by the identified candidate miRNAs. A total of 12,283,683 raw reads were obtained, with 2,623,291 unique raw reads (Table S4). After initial processing, 9,182,666 reads were obtained and could be mapped to mRNAs, corresponding to 74,918 sequences matching *G. max* genes. We identified targets for detected miRNA using the Cleaveland 3.0 pipeline. The cleaved target transcripts were categorized into five groups (categories 0, 1, 2, 3 and 4) according to the relative abundance of the tags at the target mRNA sites^33^. A total of 898 transcripts targeted by 54 miRNA families were identified (Table S5). Among these identified targets, 259 (28.8%), 38 (4.2%), 305 (34.0%), 31 (3.5%) and 265 (29.5%) targets were found to be distributed into categories ‘0’, ‘1’, ‘2’, ‘3’ and ‘4’, respectively (Table S5).

In present study, the miRNAs were predicted to cleave more than two different targets, similar with previous study^35^. For instance, gma-miR156 was predicted to slice fifteen genes belonging to the SBP family, two genes of the abscisic acid responsive element-binding factor family, two members of the global transcription factor family, one gene belonging to alpha/beta-Hydrolase superfamily and one member of the NAD(P)-binding Rossmann-fold superfamily of genes. Meanwhile, several transcripts were found to be regulated by pairs of miRNAs. For example, gma-miR156 and gma-miR395 both targeted three members (Glyma.04G081400, Glyma.13G215800 and Glyma.15G097100) of the alpha/beta-Hydrolase family, and gma-MIR1513 and gma-miR5767 sliced five genes for F-box family proteins (Table S5).

### Identification of chilling stress-responsive miRNAs in vegetable soybean

**Figure 4:**
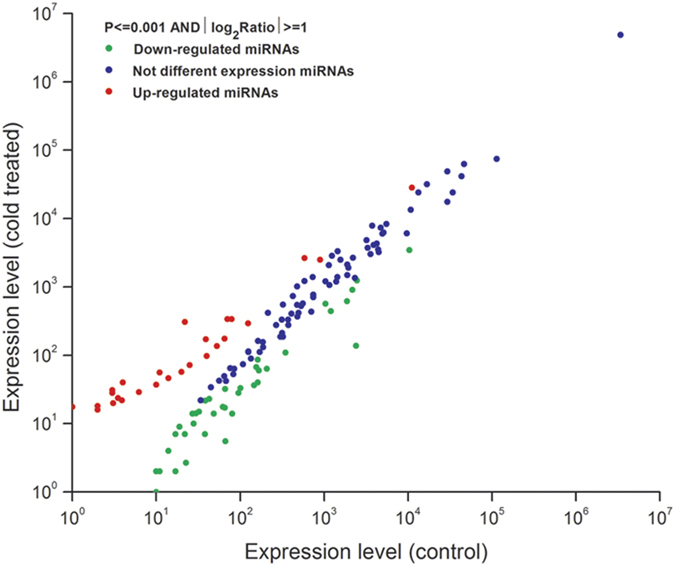
Comparison of expression patterns of miRNAs identified between chilling-stress and control libraries.

We compared the read counts from chilling-treated and control libraries to identify differential expression of miRNAs in response to chilling stress. A total of 51 miRNAs from 32 families were significantly differentially expressed between chilling-treated and control libraries (Fig. 4). Thirty and 21 miRNAs showed significant down- and up-regulation in response to chilling stress, respectively. For example, miR169c, miR169d, miR169e, miR164a and miR1507a were in higher abundance in the chilling-stress library than in the control. By contrast, miR156r, miR156a, miR159d, miR4413a, miR4413b and miR172c were down-regulated in response to chilling stress.

**Figure 5:**
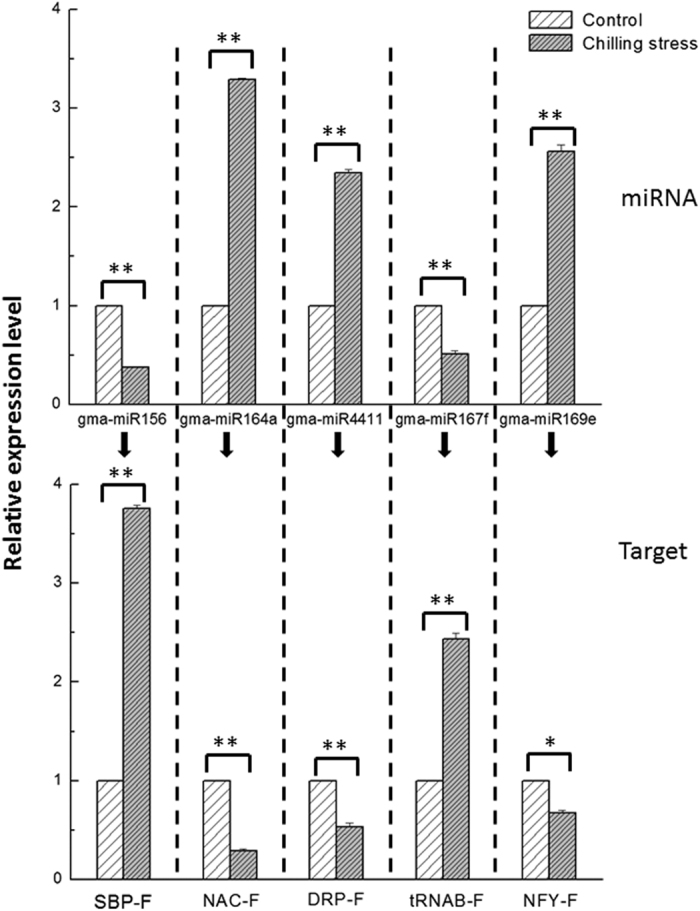
Expression profiles of miRNAs and their targets under chilling stress by qRT-PCR. All data were subjected to an analysis of variance (ANOVA) and the results presented as the mean ± SD. A *P*-value was considered to be statistically significant with (*p < 0.05) or (**p < 0.01).

The target genes were further investigated to gain insight into possible roles of the differently expressed miRNAs (Table 2). The results revealed that the potential target genes are involved in a wide range of biological processes. The differentially expressed miRNAs could be classified into four major categories. The first category included miRNA families (miR156, miR164, miR169, miR4412 and miR5374) targeting SBP, NAC, NFY, GRAS, bHLH transcription factors, respectively, which are involved in the regulation of gene expression and signal transduction. The second category included miR4411, the target gene of which is related to a disease resistance protein in the TIR-NBS-LRR class. The third category included miR5761, miR159, miR5667, miR1535, miR511, miR4382 and miR4416. Their targets play roles in adaptive responses in plant growth and development, such as hydrolase-, lyase-, transferase-, kinase-, and oxidoreductase-coding genes. The remaining miRNAs, which were less conserved and whose functions were unclear, were classified into the last category. To further validating the relationship between miRNAs and their target genes under chilling stress, we performed qRT-PCR analysis for five selected miRNAs and their targets using leaves from both chilling-treated and control vegetable soybean plants with three replicates. The results showed that miRNA-164a, miRNA-4411 and miRNA-169e were up-regulated with fold changes over 2 under chilling stress. By contrast, their target genes, *NAC*, *DRP* and *NFY* were significantly down-regulated (Fig. 5). Moreover, miRNA156a and 167f, which both exhibited low expression, also showed negative relationships with their putative targets.

**Figure 6:**
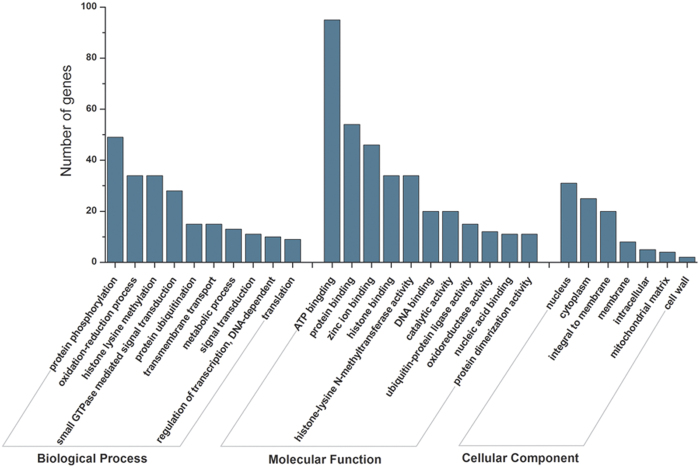
GO analysis of miRNA target genes in vegetable soybean. Only the predicted target genes for miRNAs responding to chilling stress were considered.

To better understand the functions of these differentially expressed miRNAs, we conducted gene ontology (GO) analysis of their target genes. The results demonstrated that the target genes of the differentially expressed miRNAs could be classified into 56 molecular function categories, 37 biological process categories, and 7 cell components categories (Fig. 6, Table S6). In the molecular function category, ATP binding, protein binding, zinc ion binding, histone binding, histone-lysine N-methyltransferase activity, DNA binding and catalytic activity were significantly enriched (Fig. 6). More than 35% of these miRNA targets were predicted to take part in a broad range of biological processes, such as protein phosphorylation, oxidation-reduction, histone lysine methylation, small GTPase-mediated signal transduction, protein ubiquitination, transmembrane transport, metabolic processes, signal transduction and regulation of transcription.

## Discussion

To date, a number of experiments have been conducted to discover miRNAs from grain soybean^20^,^24^. However, there were differences between grain soybean and vegetable soybean in agronomic traits^2^,^36^. Our preliminary analysis on the phylogenetic relationship between vegetable soybean and other types of soybean plants (grain soybean and wild soybean) by DNA sequencing data suggests that vegetable soybean is genetically different from both grain and wild soybean (unpublished). So, a systematic study is needed to elucidate the functions of miRNAs in response to cold stress in vegetable soybean.

### Identification of vegetable soybean microRNAs and their targets

In the present study, a total of 434 vegetable soybean miRNAs were identified (Table S1 and S2). The classical conserved miRNAs, including miR156, miR164 and miR166, were detected in vegetable soybean consistent with previous studies in grain soybean^22^,^37^. However, almost half of the detected miRNAs were not deposited in soybean miRBase in present study. The reason might be that these miRNAs are not expressed in tissues used for constructing previous small RNA libraries. For instance, most previous studies have analyzed roots from grain soybean^21^,^34^,^38^. We also identified three novel miRNAs, which were potentially generated from four different loci. However, the expression levels of novel miRNAs were much lower those that of the most known miRNAs. The results might be that the new flanking sequences were theoretically more volatile in novel miRNA than the other miRNAs^39^,^40^. The predicted lack of targets was also supported by the degradome sequencing data in our study. None of the novel miRNAs was detected to have a cleavage signature in the degradome library. We searched for putative targets of the novel miRNAs using psRNATarget software (http://plantgrn.noble.org/psRNATarget/). Interestingly, the average number of genes predicted to be targeted by the novel miRNAs was higher than that for most of the known miRNAs. Therefore, there was more vast development space for vegetable soybean miRNAs.

To gain insight into the regulatory function of miRNAs, we validating their target genes using degradome sequencing, which has been successfully applied to screen for miRNA targets in soybean and other plants^28^,^41^. In this study, a large number (898) of targets were identified and found to be distributed into five categories, which were involved in plant growth and responses to environmental changes^33^,^42^. Interestingly, we found cleavage signatures for 23 conserved and 31 non-conserved miRNA families. The result was not coincident with those of previous studies in *G. max* and *G. soja*, which found a significantly higher proportion of conserved miRNAs to have cleavage signatures^34^,^43^. It is possible that the expression patterns of the miRNAs differ in tissue/ecotype-specific and stage-specific manners, as well as in response to different environmental stresses^44^. And the degradome abundances of some targets may not be sufficient for detection in vegetable soybean leaf tissues. Moreover, the miRNA regulation mechanism also can impose translational repression on the targets, which would therefore be undetectable by degradome sequencing. The incomplete mRNA database may limit the comprehensive identification of targets as well. Consequently, further construction of small RNA and degradome libraries from different tissues and developmental stages should provide more insight into the network between miRNAs and their targets.

### Differentially expressed miRNAs under chilling stress in vegetable soybean

We reasoned that the identification of differentially expressed miRNAs would lead to a better understanding of the post-transcriptional regulation taking place in vegetable soybean under chilling stress. Accordingly, we compared the expression levels of miRNAs between chilling-treated and control samples, and classed a total of 51 differentially expressed miRNAs as chilling stress-responsive miRNAs (Table 2) with down- and up-regulation in response to chilling stress. MiR156, miR164 and miR171 were found to be significantly responsive to chilling stress in accord with those in *S. habrochaites* and *Cassava*^35^,^45^. The results indicated that some miRNAs involved in chilling stress showed consistency among several plant species. A number of responsive miRNAs showed different expression trends under chilling stress compared with those in previous reports. For instance, in previous studies, miR167 was up-regulated in response to the chilling stress in soybean nodules and miR169 was down-regulated^24^. However, the opposite trends were displayed in present study. These different regulation patterns could be due to differences between the two types of soybean species (vegetable and grain soybean) or different parts of the plant (leaf and nodule) used in the studies. In any event, the discrepancy awaits further investigation.

As expected, almost half of chilling responsive miRNAs targeted transcription factors related to regulation of plant growth and development in present study. We performed qRT-PCR to analyze the expression of the miRNAs and their targets with or without chilling stress treatment. The negative relationships between the expression of miRNAs (e.g. miR156a, miR164a, and miR169e) and their target genes (e.g. SBP-F, NAC-F, and NFY-F) suggest that miRNAs and miRNA-mediated molecular pathways modulate responses to chilling stress in vegetable soybean. SPL, which was targeted by miR156, was proved to participate in plant development^35^. NAC, targeted by miR164, was responsive to abiotic stress in plants^46^,^47^,^48^. Surprisingly, several low temperature-induced transcription factor genes, such as genes encoding CBF, WRKY and MYB were not detected in the present study, perhaps because these genes were not expressed at the time of sampling or in the leaves.

It is worth noting that only one-thirds of the chilling responsive miRNAs were non-conserved in the present study. These results differ from those of a previous study in which the non-conserved miRNAs accounted for a larger share of the total miRNAs responsive to abiotic stress in soybean^34^. The difference in the proportion of non-conserved miRNAs might be due to the fast development of high-throughput sequencing, which may lead to classification of some precursor non-conserved miRNAs as conserved miRNAs. Interestingly, the novel miRNAs identified herein, such as gma-miRN1 and gma-miRN3, were notably more expressed under chilling stress, although their expression levels were lower than those of other conserved miRNAs. Our *in silico* analysis identified a set of predicted target genes, including those for glycerol-3-phosphate acyltransferase and L-ascorbate oxidase, which play an important role in regulation of the pathway responsive to chilling stress. These results thus provide new clues for future research on miRNAs under this abiotic stress.

## Conclusions

In conclusion, our study provides the first results pointing to miRNA regulation of gene expression under chilling stress in vegetable soybean leaves. We applied the degradome sequencing approach to detect hundreds of cleaved targets, which revealed the interaction between miRNAs and their targets in vegetable soybean. Although the foundation of the complex miRNA-mediated regulatory networks remains to be unraveled, this miRNA dataset represents an important supplement to the soybean miRNA database and should be useful to understand the gene regulatory networks in vegetable soybean and other species. We also discovered 51 chilling-responsive miRNAs, which serves as a good start to understanding the complex gene regulatory function of miRNAs in vegetable soybean under chilling stress.

## Methods

### Plant materials

The experiments were conducted in the Institute of Vegetables, Zhejiang Academy of Agricultural Sciences. Vegetable soybean ‘Taiwan 75’ was planted in pots at a photosynthetic photon flux density (PPFD) of 600 μmol m^−2^ s^−1^ with a photoperiod of 12 h, temperatures of 25 °C (day) and 17 °C (night), and a humidity between 50% and 85%. At the one true-leaf stage, the seedlings were divided into two groups for treatment: one group was exposed to 4 °C for 24 h as chilling treatment (Chilling stress), while the other group was not treated with chilling stress (Control).

### Sample preparation and total RNA extraction

Leaves of three replicates with 24 h chilling treatment and control were collected, respectively, and stored at −80 °C. Total RNA from each replicate was extracted separately with TRIzol reagent (Invitrogen, USA) according to the manufacturer’s instructions. RQ1 RNase-Free DNase (Promega, USA) was used to remove genomic DNA contamination. The total RNA quantity and purity were analysis by Bioanalyzer 2100 and RNA 6000 Nano LabChip Kit (Agilent, CA, USA).

### Small RNA library construction and Illumina sequencing

For Solexa sequencing, the three RNA samples of equal amount for chilling treatment and control were then pooled together for expression profiling, respectively. About 1 μg of total RNA were used to construct small RNA library following the protocol of TruSeq Small RNA Sample Prep Kits (Illumina, San Diego, USA). As a result, two small-RNA libraries, corresponding to the conditions of chilling stress and control, were constructed. Then we performed the single-end sequencing on an Illumina Hiseq2500 at the LC-BIO (Hangzhou, China) following a recommended protocol^49^.

### Identification of known and novel miRNAs

To identify known and novel miRNAs, a data processing procedure was conducted with some modification suit for plant in hairpin prediction provided by LC Sciences Service^34^,^47^. Briefly, all of the raw reads were firstly subjected to the Illumina pipeline filter, and then processed by ACGT101-miR program (LC Sciences, Houston, Texas, USA) to filter out adapter sequences, low-quality and low copy reads, as well as common RNA families (rRNA, tRNA, snRNA, snoRNA). Subsequently, filtered sequences with 18–25 nt in length were compared to miRNA database, miRBase 20.0 (http://www.mirbase.org/) by BLASTn search to identify the known miRNAs. Length variation at both 3′ and 5′ ends and one mismatch inside of the sequence were allowed in the alignment. The unique sequences mapping to specific species mature miRNAs in hairpin arms were identied as known miRNAs. The unique sequences mapping to the other arm of known specific species precursor hairpin opposite to the annotated mature miRNA-containing arm were considered to be novel 5p’ or 3p’ derived miRNA candidates. The remaining sequences were mapped to other selected species precursors (with the exclusion of specific species) in miRBase 20.0 by BLAST search, and the mapped pre-miRNAs were further blasted against the specific species genomes to determine their genomic locations. The above two were defined as known miRNAs.

Then, the unmapped sequences were subjected to blast against the soybean genome to identify novel miRNAs and predicated stem-loop hairpin secondary structures by using RNAfold software (http://rna.tbi.univie.ac.at/cgi-bin/RNAfold.cgi). The criteria for secondary structure prediction was as described by previous studies^47^, including: (1) the miRNA located in one arm of the folded stem-loop hairpin secondary structure, (2) less than 12 nt in one bulge and more than 16 base pairs in the stem region of the predicted hairpin, (3) less than 20 nt in length of hairpin loop and more than 50 nt in length of total hairpin, (4) no more than 4 nt and two biased errors in one bulge, (5) less than two biased bulges and 4 mismatches, as well as more than 12 base pairs in mature region, (6) more than 80% of mature region in stem, (7) predicted secondary structure had higher negative minimal folding energy (MFE) and minimal free folding energy indexes (MFEI).

### Expression pattern of miRNAs between chilling stress and control libraries

To investigate the differentially expressed miRNAs between chilling stress and control libraries, each identified miRNAs read count was normalized to the total number of miRNA reads in each given sample and multiplied by a million (RPKM). The *p* value (≤0.001) and absolute value of log_2_Ratio (≥1), indicating the ratio of RPKM values for the chilling treated and control libraries, were used to judge the significance of gene expression differences^48^.

### Degradome library construction and target identification

A degradome library was constructed from vegetable soybean leaves to predict the potential target mRNAs following the methods described previously by German *et al.*^50^. The purified cDNA library was used for cluster generation on Illumina’s Cluster Station and then sequenced on Illumina Hiseq 2500 following vendor’s instruction for running the instrument. Raw sequencing reads were obtained using Illumina’s Pipeline v1.5 software. Identification and classification of categories of the sliced miRNA targets were processed according to the CleaveLand 3.0 pipeline^27^. The sliced-target transcripts were grouped into five categories. Category ‘0’ is defined as >1 raw read at the position, with abundance at a position equal to the maximum on the transcript, and with only one maximum on the transcript. Category ‘1’ is described as >1 raw read at the position, with abundance at the position equal to the maximum on the transcript, and more than one maximum position on the transcript. Category ‘2’ includes >1 raw read at the position, and abundance at the position less than the maximum but higher than the median for the transcript. Category ‘3’ comprised the transcripts with >1 raw read at the position, and abundance at the position equal to or less than the median for the transcript; and category ‘4’ showed only one raw read at the position. All of the candidate target transcripts were performed to gene ontology (GO) analysis by using the AgriGO toolkit^51^.

### Verification of miRNAs and their targets

To validate the expression of the identified miRNAs and their targets, miRNAs and their target genes with different expression patterns were randomly selected for qRT-PCR. The analysis was performed using 24 h chilling treatment and control plants for RNA extraction. Thirty-five miRNAs (Table S7) were polyadenylated and reverse transcribed by using One Step PrimeScript^®^ miRNA cDNA Synthesis Kit (Takara, China) according to the manufacturer’s protocol in triplicate for each sample^48^. The PCR reactions were performed in ABI Step One (Applied Biosystems, USA) using Platinum SYBR Green qPCR SuperMix-UDG (Invitrogen, USA). PCR cycling began with template denaturation and hot start Taq activation at 95 °C for 2 min, then 40 cycles of 95 °C for 15 sec, and 60 °C extension step. The U6 snRNA was used as an internal reference gene for normalization (Table S7). For target genes, the extracted RNAs were reverse-transcribed by using specific primers, and *GmACT11* was used as an internal reference gene for normalization (Table S8). The qRT-PCR reaction was performed according the manufacturer’s protocol with three biological replicates^52^. All of the miRNA and target gene sample cycle threshold (Ct) values were standardized and the 2^–∆∆CT^ method was used to analyse the relative changes in expression from real-time qPCR experiments^53^. All data were subjected to an analysis of variance (ANOVA) and the results presented as the mean ± SD. A *P*-value was considered to be statistically significant with (*p < 0.05) or (**p < 0.01).

## Additional Information

**How to cite this article**: Xu, S. *et al.* Identification of chilling-responsive microRNAs and their targets in vegetable soybean (*Glycine max* L.). *Sci. Rep.* **6**, 26619; doi: 10.1038/srep26619 (2016).

## Supplementary Material

Supplementary Figures

Supplementary Tables

## Figures and Tables

**Table 1:** Read abundance of various classes of small RNAs in control and chilling-treated vegetable soybean.

RNA class	Control		Chilling	
	Raw reads	Unique reads	Raw reads	Unique reads
Total	8,944,338	1,376,736	10,723,343	1,621,746
3ADT&length filter	1,043,443	550,363	1,125,782	529,468
Junk reads	11,683	6,174	15,161	8,421
Rfam	538,845	54,634	478,694	47,744
mRNA	1,145,459	198,715	1,120,715	200,375
Repeats	4,378	1,835	3,553	1,632
rRNA	383,616	36,183	327,010	31,240
tRNA	134,229	12,847	133,543	11,724
snoRNA	1,644	927	1,226	767
snRNA	1,320	851	1,079	740
Other Rfam RNA	18,036	3,826	15,836	3,273
Clean reads	6,559,098	592,392	8,273,245	857,426

**Table 2:** Differentially expressed miRNAs identified in chilling-treated vegetable soybean compared with control.

miRNA	miRNA sequence	normal reads (control)	normal reads (chilling -treated)	*log^2^ (chilling -treated/control)	putative target
gma-miR156r	CTGACAGAAGATAGAGAGCAT	2580.30	127.89	−4.33	SPL/SBP
gma-miR5761a	TTTTGTGTCGTGAAGCTTTTG	71.43	5.12	−3.80	homogentisate phytyltransferase 1, cyclophilin71
gma-MIR5032-p3	AACGGAGCCACTGTGAAGAAGT	10.74	0.93	−3.53	
gma-miR159d	AGCTGCTTAGCTATGGATCCC	24.35	2.48	−3.29	PDH-E1 ALPHA
gma-miR172-5p	GTAGCATCATCAAGATTCACA	86.38	13.03	−2.73	
gma-MIR171e-p5	TGTTGGACGGTTCAATCAAA	11.82	1.86	−2.67	
gma-MIR4413a-p3	ATACAGTGACTTACAATTCTC	40.82	6.52	−2.65	
gma-MIR319i-p5	AGGAGCTTCCTTCAGCCCATG	10.74	1.86	−2.53	Mitochondrial import inner membrane translocase subunit, Chaperone protein htpG family protein
gma-MIR5372-p3	ATAATGTACAACACAATTATC	174.02	37.24	−2.22	
gma-MIR159d-p3	CTTCCATATCTGGGGAGCTTC	156.65	33.82	−2.21	pyruvate dehydrogenase E1 alpha
gma-miR4413b	TAAGAGAATTGTAAGTCA	70.36	15.83	−2.15	RNA processing factor 2, PPR superfamily protein, TPR-like superfamily protein, WRKY35
gma-miR172c	AGAATCTTGATGATGCTGCAN	66.96	16.29	−2.04	
gma-miR5674a	TAATTGTGTTGTACATTATCA	52.10	13.03	−2.00	ATP binding, NOP56-like pre RNA processing ribonucleoprotein, PPR superfamily protein
gma-MIR1508a	ACTGCTATTCCCATTTCTAAA	222.35	58.65	−1.92	ATP binding, NOP56-like pre RNA processing ribonucleoprotein, Pentatricopeptide repeat (PPR) superfamily protein, peroxisomal ABC transporter 1, rna processing factor 2, Tetratricopeptide repeat (TPR)-like superfamily protein
gma-MIR167g	GATCATGTGGCTGCTTCACC	371.66	101.47	−1.87	
gma-MIR167f	AGATCATGTGGCAGTTTCACC	23.63	6.52	−1.86	
gma-MIR4416c	ACGGGTCGCTCTCACCTGGAG	2016.22	572.53	−1.82	
gma-miR156a	TGACAGAAGAGAGTGAGCAC	11137.16	3193.80	−1.80	SPL/SBP
gma-miR394a-3p	AGCTCTGTTGGCTACACTTTG	30.08	9.31	−1.69	
gma-MIR4412	GGCGTAGATCCCCACAACAGT	18.26	6.52	−1.49	GRAS family transcription factor
gma-miR4413a	TAAGAGAATTGTAAGTCACTG	2328.27	838.79	−1.47	GBA2 type family protein, PPR superfamily protein, rna processing factor 2, TPR-like superfamily protein
gma-miR172h-5p	GCAGCAGCATCAAGATTCACA	167.03	62.37	−1.42	ADP-ribosylation factor A1F, membrane steroid binding protein 1, Ribosomal protein L12
gma-MIR1535a-p5	AGACATCACCACAAACAAGTC	20.41	8.38	−1.28	Late embryogenesis abundant (LEA) hydroxyproline-rich glycoprotein family, Transducin/WD40 repeat-like protein
gma-miR393a	TTCCAAAGGGATCGCATTGATC	31.92	13.13	−1.28	
gma-miR5667	AAACAGATCTAAATGGATTCC	70.90	29.79	−1.25	nuclear encoded CLP protease 5
gma-miR397b-3p	TATTGACGCTGCACTCAATCA	2641.39	1150.65	−1.20	
gma-MIR5374-p3	TTCGAATGTCAGATTATAAAA	29.00	13.03	−1.15	bHLH DNA-binding superfamily protein
gma-miR162a	TCGATAAACCTCTGCATCCAG	173.48	80.06	−1.12	
gma-miR5767	TGGAGGACCTTTGAAGGTGCA	1116.07	524.12	−1.09	F-box family protein
gma-miR171n	TTGAGCCGCGTCAATATCTTA	41.36	20.25	−1.03	
gma-miR1535a	CTTGTTTGTGGTGATGTCTAG	133.73	272.30	1.03	isopentenyltransferase 5, Transducin/WD40 repeat-like protein
gma-MIR166r-p5	GGAATGCAGTGTGGTCCAAGG	42.97	90.30	1.07	
gma-miR1507a	TCTCATTCCATACATCGTCTGA	11988.30	26404.05	1.14	PIF1 helicase, Eukaryotic aspartyl protease family protein, NFY
gma-MIR1511-p5	GTGGTATCAGGTCCTGCTTCA	56.93	126.61	1.15	methionine sulfoxide reductase B 1
gma-MIR1520k-p3	TTGACATCCAATCAGAACATGACA	70.18	162.53	1.21	Tesmin/TSO1-like CXC domain-containing protein
gma-miR4376-5p	TACGCAGGAGAGATGACGCTG	962.46	2311.55	1.26	
gma-miR4411	TTATTGTAACTAATTTGTCGGT	15.04	42.82	1.51	Exostosin family protein
gma-miR4382	TATGTTAACTGATTTCATGGAT	10.74	34.45	1.68	SGNH hydrolase-type esterase superfamily protein, Pyridine nucleotide-disulphide oxidoreductase family protein
gma-MIR166e-p5	GGAATGTTGGCTGGCTCGAGG	42.03	158.78	1.92	
gma-miR164a	TGGAGAAGCAGGGCACGTGCA	627.16	2452.21	1.97	NAC domain containing protein 1/100, LisH dimerisation motif;WD40/YVTN repeat-like-containing domain
gma-miR171b-3p	CGAGCCGAATCAATATCACTC	6.71	27.00	2.01	
gma-miR4416b	TGGGTGAGAGAAACGCGTATC	75.73	313.11	2.05	Malectin/receptor-like protein kinase family protein
gma-miR5559	TACTTGGTGAATTGTTGGATC	11.82	52.13	2.14	
gma-miR169e	TGAGCCAAGGATGACTTGCCG	4.19	20.37	2.28	
gma-miR169c	AAGCCAAGGATGACTTGCCGA	3.76	21.96	2.55	nuclear factor Y, subunit A1
gma-MIR169a-p3	GGCAAGTTGTGTTTGGCTAT	2.15	14.90	2.79	ATP binding microtubule motor family protein, DNAse I-like superfamily protein
gma-MIR171b-p5	CGTGATATTGGTACGGCTCATC	3.22	26.07	3.02	
gma-MIR156l-p3	GCTCTCTAAGCTTCTGTCATCC	4.30	37.24	3.12	nuclear factor Y, subunit B13
gma-MIR156m-p3	GCTCTCTAGGCTTCTGTCATCC	3.22	28.86	3.16	
gma-miR169l-3p	CGGGCAAGTTGTTTTTGGCTAC	1.07	16.29	3.92	C3HC4 type (RING finger) family protein
gma-miR169d	TGAGCCAAGGATGACTTGCCG	0.97	17.42	4.16	nuclear factor Y, subunit A6, jasmonate-zim-domain protein 3

^*^Log^2^ ratio of normalized miRNA expression in chilling treatment compared with control.
