# Supplementary material for: Identification of chilling-responsive microRNAs and their targets in vegetable soybean (Glycine max L.)
Source: Sci Rep. 2016 May 24;6:26619. doi: 10.1038/srep26619 (PMC4877674; doi:10.1038/srep26619)
Supplement: Supplementary Figures [file srep26619-s1.pdf]

Title:

Identification of chilling-responsive microRNAs and their targets in vegetable soybean (*Glycine max* L.)

Author list:

Shengchun Xu[1, †,*], Na Liu[1, †], Weihua Mao[2], Qizan Hu[1], Guofu Wang[3], Yaming Gong[1,*]

Supplementary figure legends

Figure S1. Effect of chilling stress on relative growth rate (RGR) and MDA content under different chilling periods. All data were subjected to an analysis of variance (ANOVA) and the results presented as the mean±SD. A *P*-value was considered to be statistically significant with * (p<0.05) or ** (p<0.01).

Figure S2. Coefficient analysis between miRNA expression ratios obtained from the miRNA-sequencing and the qRT-PCR data.

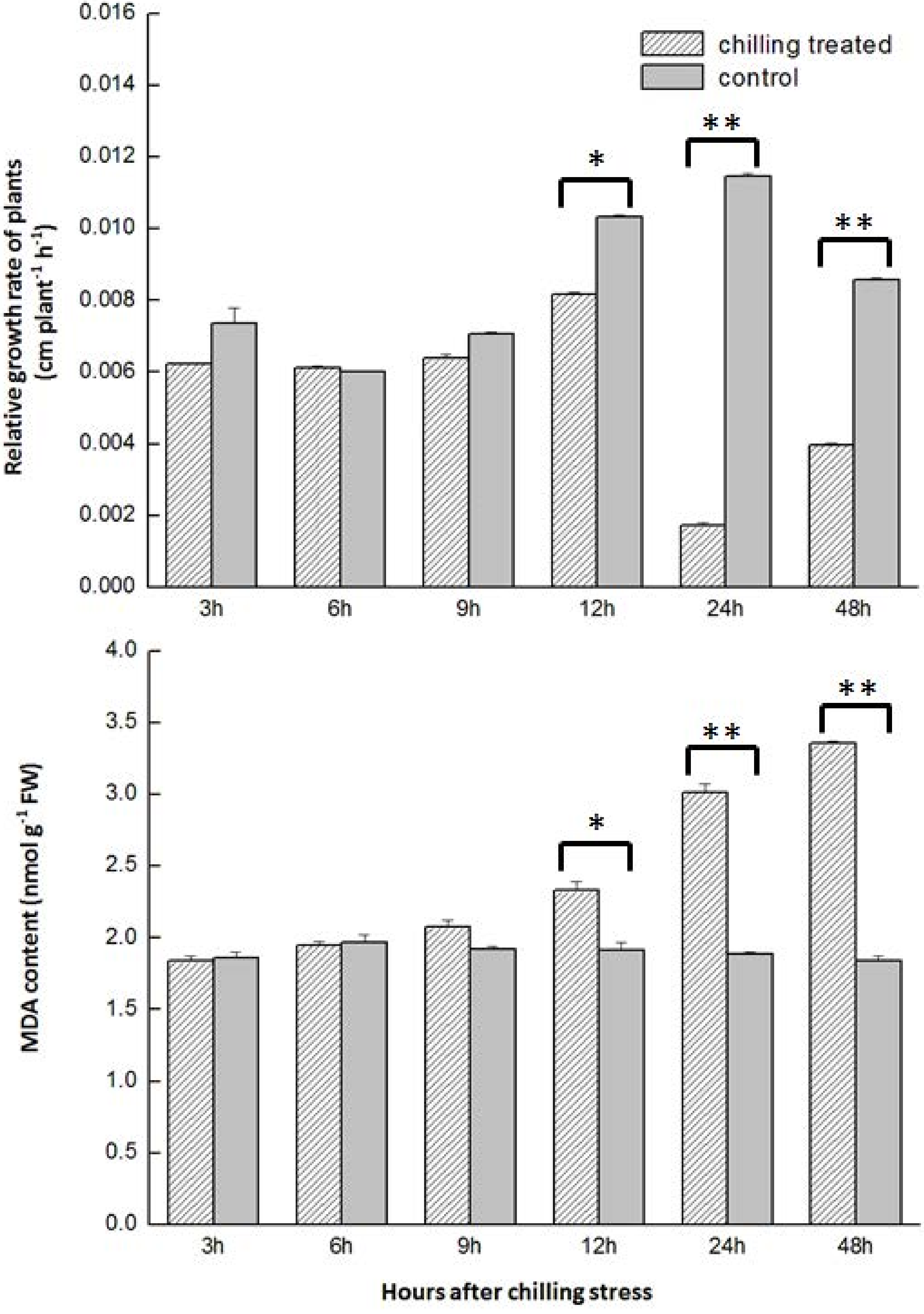

chilling treated
control
Relative growth rate of plants (cm plant-1 h-1)
0.016
0.014
0.012
0.010
0.008
0.006
0.004
0.002
0.000
*
**
**
3h
6h
9h
12h
24h
48h
MDA content (nmol g-1 FW)
4.0
3.5
3.0
2.5
2.0
1.5
1.0
0.5
0.0
*
**
**
3h
6h
9h
12h
24h
48h
Hours after chilling stress

Figure S1

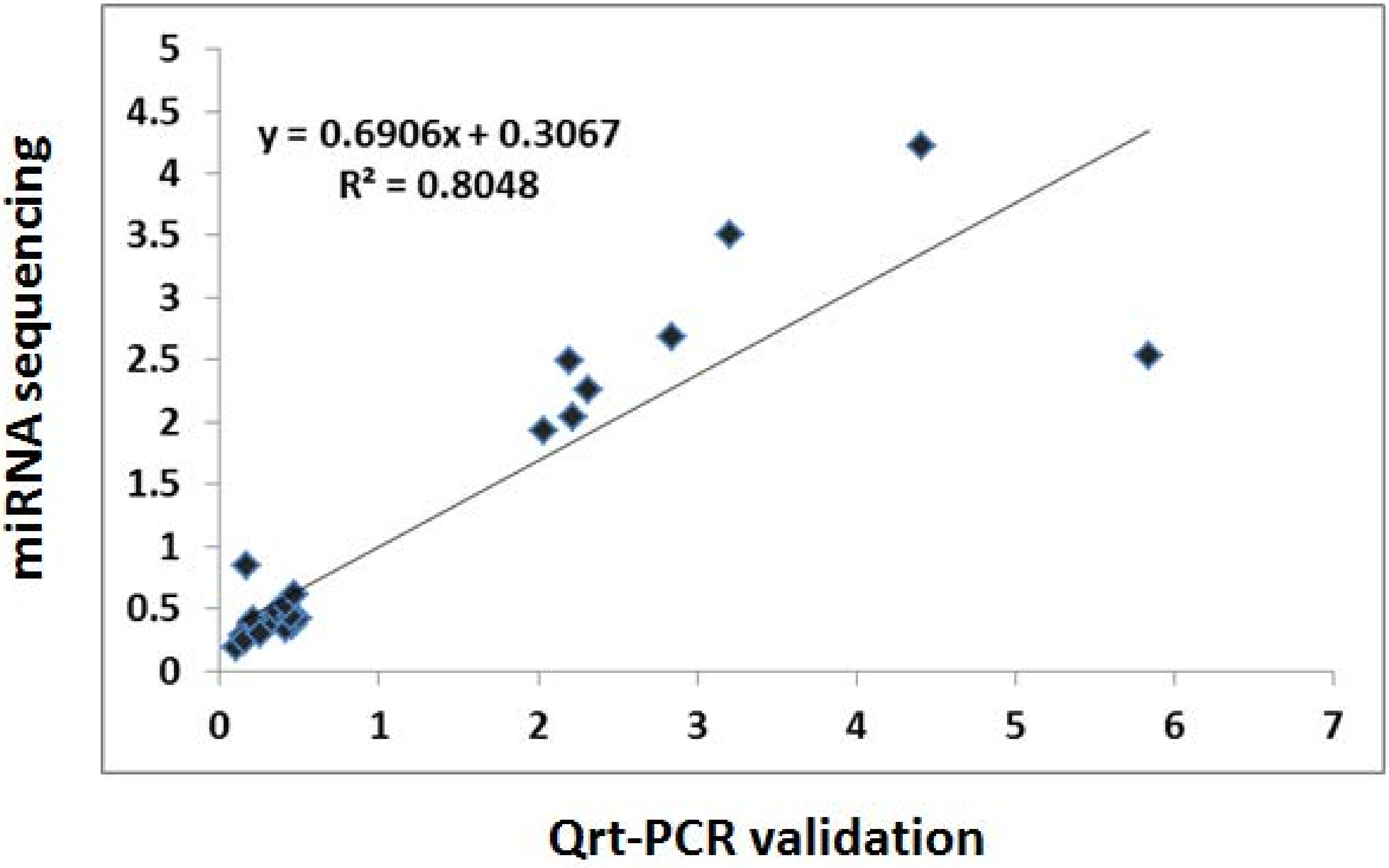

y = 0.6906x + 0.3067
R² = 0.8048
miRNA sequencing
0
0.5
1
1.5
2
2.5
3
3.5
4
4.5
5
0
1
2
3
4
5
6
7
Qrt-PCR validation

Figure S2
